# MUSICIAN’S DYSTONIA IN A PERCUSSIONIST – CLINICAL VIDEO ANALYSIS AND BOTULINUM TOXIN INTERVENTION: A CASE REPORT

**DOI:** 10.2340/jrm.v56.34877

**Published:** 2024-03-14

**Authors:** Tomás CAROÇO, Ana ZÃO, Júlia RIBEIRO, Ana N. FIALHO, Victor MILET, Bruna MEIRA

**Affiliations:** 1 Physical and Rehabilitation Medicine Department, Hospital de Faro, Unidade Local de Saúde do Algarve, Faro; 2 Algarve Biomedical Center Research Institute (ABC-RI), Faro; 3 Chronic Pain Unit and Physical and Rehabilitation Medicine Department, Hospital de Santo António, Unidade Local de Saúde de Santo António, Oporto; 4 Faculty of Medicine, University of Porto (FMUP), Oporto; 5 Center for Health Technology and Services Research and Health Research Network (CINTESIS@RISE), Oporto; 6 International Center of Arts Medicine, CUF Porto Institute, Oporto; 7 Physical and Rehabilitation Department, Hospital de Portimão, Unidade Local de Saúde do Algarve, Portimão; 8 Neurology Department, Hospital de Egas Moniz, Unidade Local de Saúde Lisboa Ocidental, Lisboa, Portugal

**Keywords:** botulinum toxin, case report, dystonia, focal, task-specific, electromyography, music, ultrasonography

## Abstract

**Objective:**

Musician’s focal hand dystonia is a painless task-specific focal dystonia, which presents with involuntary movements, abnormal postures, and loss of fine motor dexterity. We report here the case of a 63-year-old male, percussionist, with african ethnicity, with musician’s focal hand dystonia who was treated with botulinum toxin, and describe the results at 4-weeks follow up.

**Methods:**

Clinical examination and video analysis revealed abnormal flexion of the 3^rd^ finger, followed by flexion of the 4^th^ and 5^th^ fingers while playing the congas. Based on these findings, a diagnosis of musician's focal hand dystonia was established. Ten units of botulinum toxin were injected into the muscle fibres of the flexor digitorum superficialis corresponding to the 4^th^ finger using electromyography and ultrasound guidance. Four weeks later, the patient reported a subjective 60% improvement in his performance. He emphasized the effect of botulinum toxin on performance speed and tension over the forearm and hand.

**Conclusion:**

Botulinum toxin is not a definitive treatment for musician’s focal hand dystonia, but it may potentiate other definitive rehabilitation techniques. More research is needed to determine the long-term effects of botulinum toxin on function enhancement in musician’s focal hand dystonia.

LAY ABSTRACT

Musician’s focal hand dystonia is a pathology that affects musicians, causing unintentional movements that can impact musical performance. This work reports a case of a male percussionist who experienced musician’s focal hand dystonia. Distinguishing dystonic movements from compensatory movements that attempt to control the hand is often challenging. Video analysis allows us to differentiate the dystonic movements from the compensatory movements. Botulinum toxin is a medication that, when injected into a muscle, induces weakness. This weakness can be beneficial if the toxin is injected into a dystonic muscle. However, it is important to note that the root cause of dystonia lies in the brain, and botulinum toxin serves as a facilitator for other rehabilitation techniques. These techniques enable a modification of the brain circuits responsible for dystonia. Further research is necessary to elucidate the role of botulinum toxin in the treatment of musician’s focal hand dystonia. This case highlights a successful example of the use of botulinum toxin in musicians with focal hand dystonia and the importance of a careful analysis of the dystonic phenomenology to achieve a good response with toxin injections.

Dystonia is a movement disorder characterized by sustained or intermittent muscle contractions causing abnormal, often repetitive, movements, postures, or both. Dystonic movements are typically patterned, twisting or tremulous (1).

Musician’s focal hand dystonia (MFHD) is a painless task-specific focal dystonia, presenting with involuntary movements, abnormal postures, and loss of fine motor dexterity (2, 3). MFHD affects approximately 1–2% of professional musicians during their career (4) and MFHD accounts for 5% of all dystonia patients (the worldwide estimated prevalence of primary dystonia is 16.4 per 100,000 persons) (5). The real prevalence is likely to be even higher. MFHD affects learned technical bimanual movements, predominantly in the context of playing a musical instrument. The limb that is subject to the greatest fine motor demands is the one most affected (2–4). Learning a musical instrument requires brain plasticity, and musician’s dystonia results from an exaggeration of the changes that are required to attain musicianship at a professional standard. MFHD is associated with prolonged practice, usually at the peak of a musician’s career. Typically, MFHD is painless, although muscle ache has been reported with prolonged spasms. Symptoms in percussionists may develop as episodic finger curling. Most musicians will attribute these subtle imperfections to poor practice, and proceed to augment their efforts with more repetition, which often exacerbates the symptoms (6).

Sensory tricks are strategies used by patients with MFHD to overcome difficulties performing. These tricks involve changes in playing technique or local sensory stimulation that lead to symptom softening. According to Llobet & Molas (8), sensory tricks may be: “purely sensorial; changing the execution; limitations in playing; combinations of the former”. Since dystonia results from an interaction between sensorial and motor malfunctioning, sensory tricks rely on sensory or motor input to force the neuronal circuit to execute the correct movement or posture (7). Purely sensorial tricks rely on a specific sensory input; for example, contact with an object, without having a mechanical action on the musicians’ bodies (8). Changing the execution may be beneficial to the performance of a musician with MFHD. A famous example is Robert Schumann, who composed a piece (Toccata op.7) avoiding the use of the 3^rd^ finger in virtuoso passages (9).

The use of semi-rigid materials is quite common among musicians with dystonia. Although the material may be effective by opposing the dystonic movement, it acts more as a proprioception modifier (8).

Botulinum toxin (BoNT-A) may be a safe and effective treatment for MFHD, as it is for other types of focal dystonia (10). Clinical evaluation of therapeutic intervention in these patients requires the use of a measure-ment instrument in the clinic. Specific measure-ment of the movements involved in the dystonic limb is crucial in muscle selection for BoNT-A injection. Often, video recordings of the musician performing are necessary to inform clinical analysis (6). BoNT-A injections in patients with MFHD may be deleterious, causing weakness in muscle groups that are crucial to musical performance.

A literature search (PubMed) was performed with the following keywords: “percussionist” and “dystonia”. This yielded 5 results. This case report represents a characteristic presentation of MFHD with an identifiable cause of the dystonic pattern and videographic documentation of the patient’s performance and effects of BoNT-A injection.

We describe here a case of a percussionist with MFHD; explain the importance of performance examination and video analysis; detail the administration of BoNT-A and present the results (objective and subjective) at 4-weeks follow-up. Finally, we discuss the importance of reporting the results of BoNT-A injections of patients with MFHD in order to develop guidelines.

## CASE REPORT

A man in his 60s, a musician who plays drums and congas, sought evaluation in a physical and rehabilitation medicine and neurology consultation. He reported involuntary movements of the left-hand fingers. Specifically, he experienced difficulty extending his fingers properly while playing the congas. However, he reported no deficits in playing the drums. The symptoms first appeared 7 years previously during a stressful period when he also reported having mechanical left epicondyle pain. Seeking medical attention, he consulted an orthopaedic surgeon and underwent shockwave therapy, which successfully treated the pain. Despite this, involuntary movements persisted, affecting his musical performance. The patient noted that the symptoms temporarily improved with alcohol consumption and were not correlated with periods of increased musical activity. To alleviate the involuntary flexion of the fingers, the patient used adhesive tape to maintain the 4^th^ finger in extension and crossing the 3^rd^ finger over the 2^nd^ finger (Fig. S5).

Subsequently, the patient was examined at a second orthopaedic surgery consultation, where he received a diagnosis of stenosing tenosynovitis (trigger finger) of the 4^th^ finger. Peritendinous glucocorticoid injection was provided, but did not provide relief. Following these unsuccessful interventions, the patient attended neurology and physical and rehabilitation medicine consultations.

During the neurological examination, involuntary movements of the left-hand fingers were observed: flexion of the 4^th^ finger involving metacarpophalangeal articulation, with extension of the interphalangeal; flexion of the 3^rd^ finger involving both metacarpophalangeal and interphalangeal articulation; extension of the 2^nd^ finger’s metacarpophalangeal articulation with inter-phalangeal flexion; and cubital wrist deviation. The remainder of the examination was otherwise normal.

The patient was then assessed in a musical studio, where his performance on the congas and drums was recorded. Video analysis of the congas performance in slow motion revealed initial flexion of the 3^rd^ finger, followed by flexion of the 4^th^ and 5^th^ fingers (Fig. S1). Conversely, no anomalies were detected in the video analysis of the drum performance (Fig. S2).

In collaboration with neurology, a diagnosis of MFHD was established. A therapeutic trial with Trihexyphenidyl 2 mg twice-daily and levodopa-carbidopa was undertaken, without benefit.

After explaining the procedure to the patient, botulinum toxin was administered. Ten units of Botox^®^ (Abbvie, USA) were injected into the muscle fibres of the flexor digitorum superficialis (FDS) corresponding to the 4^th^ finger, using dual guidance (handheld electromyography (EMG) and ultrasound (US)) (Fig. S3). In the examination and video analysis 4 weeks later, the patient reported a subjective 60% improvement in his performance ability. He emphasized the effect of BoNT-A on performance speed and tension over the forearm and hand. He reported that the benefits, after 6 weeks, started to fade until reinjection at the 12^th^ week (Fig. S4).

## DISCUSSION

This case of MFHD highlights the importance of a careful and thorough examination and video analysis to better evaluate the phenomenology of involuntary dystonic movements. Such assessment is crucial for selecting the muscles for BoNT injection in this condition. BoNT treatment can have a positive response while minimizing side-effects and muscle weakness, enabling patients to maintain their professional activity.

The late diagnosis of MFHD in the current patient aligns with findings by Llobet & Molas (8), who noted that such patients often receive misdiagnoses of other clinical conditions. Diagnosis is frequently delayed and tendon disease or overuse is initially suspected. Llobet & Molas (8) described that patients with MFHD often attend neurology and orthopaedic consultations. The most frequent diagnosis from orthopaedic surgeons is nerve compression (23.2%; *n* = 69). However, 70.3% (*n* = 54) of neurologists reach the correct diagnosis (7). In the current case, the diagnosis was made by neurology and physical and rehabilitation medicine clinicians.

Emerging evidence suggests a connection between motor and sensory functions in dystonia pathophysiology. However, it remains unclear whether motor or sensorial dysfunction is the primary cause of dystonia (7). In the current case, the patient’s initial symptom was left epicondyle pain and stress. The literature indicates that pain and stress can act as triggers for MFHD in individuals who might already be predisposed towards this condition. It suggests that an unpleasant sensorial experience, such as pain, may be implicated in the development of MFHD. Pain causes hyperactivity/sensitization, to inhibit movement and protect injured tissues. This sensitization depends on a tremendous plasticity (11). While increased cerebral plasticity is typically beneficial in neurorehabilitation, in musicians, heightened cerebral plasticity and ease of motor and sensory map reorganization may contribute to the predisposition to dystonia (7, 12).

It is notable that the current patient developed 2 sensory tricks. The use of tape not only opposes the dystonic movement, but also may provide a proprioceptive input that might be beneficial to the musician (Fig. S5) (8). In addition, he reported that crossing the 3^rd^ finger over the 2^nd^ finger resulted in a reduction in symptoms (Fig. S6). This latter sensory trick not only serves as a mechanical opposition to the dystonic posture, but also suggests the 2^nd^ finger flexor fibers are healthy and, apparently, not affected by dystonia. Sensory tricks can offer valuable clues during examination and in the final diagnosis, shedding light on a condition that affects motor patterns rather than only muscles.

According to the literature, video analysis is a major step in the process of selection of muscles for BoNT-A injection (6). Video analysis aids in differentiating dystonic muscle fibres within the movement. In this case, video analysis revealed that the 3^rd^ and 4^th^ finger flexor muscle fibres were the first to be directly affected by dystonia (Fig. S1). Subsequently, the 5^th^ finger adopted a dystonic posture. The 2^nd^ finger adopted an unsuccessfully compensatory extension gesture. An earlier evaluation might have provided a clearer distinction between the affected fingers. We decided to inject the FDS. There is no guideline regarding the muscle selection process in MFHD (6). Considering the dystonic posture of flexion of the metacarpophalangeal, proximal and distal interphalangeal joints, we might have also injected the flexor digitorum profundus (FDP). Developing guidelines for muscle selection based on specific dystonia patterns in various instrumentalists would be a significant achievement. This necessitates more case reports similar to this one and a comprehensive final analysis.

Dual guidance (EMG and US) allowed the dystonic fibres to be detected. With a handheld EMG, a characteristic dystonic signal (a tonic discharge) was detected within the 4^th^ finger fibres of the FDS (Fig. S3) (13, 14). It is important to note that this type of EMG device does not allow for the analysis of frequency and amplitude of the signal. The study should also have explored the EMG signal of the 3^rd^ finger corresponding FDS fibres and the FDP fibres, which could have provided a more comprehensive assessment (6).

MFHD often follows a typical finger distribution, with affected fingers typically being ulnar to a primary affected finger. This principle supported the injection of the FDS fibres corresponding to the 3^rd^ finger.

The detection of characteristic EMG dystonic signal within a muscle without task performing suggests that MFHD has lost its specificity, as described in the literature (15). This patient had isolated dystonia, with a typical MFHD evolution, and no other neurological findings. The fact that the patient is also a drum player suggests MFHD aetiology. Indeed, drum playing involves 4^th^ and 5^th^ finger flexion to produce drumstick swing (Fig. S2). The repetitive drumstick swing technique might have contributed to alterations in the sensorial organization and sensorial-motor integration described in MFHD pathophysiology (7).

The patient described pain and tension over the extensor compartment of the left forearm after long periods of playing the congas. This is suggestive of overuse syndrome of the wrist and finger extensors, which attempt to compensate for the dystonic posture.

BoNT-A does not enhance sensorial-motor integration, which is the foundational aspect of MFHD. Therefore, other neurorehabilitation techniques must be considered in MFHD, such as patient education and sensory motor retuning (SMR). Patient education relies on explaining the physiopathology of MFHD, to decrease tension, compensatory movements, and MFHD progression. SMR aims to reorganize the cerebral cortical map of sensorial and motor function through rehabilitation with the musical instrument. SMR is based on constraint-induced movement therapy. Compensatory fingers are splinted, focusing the musician’s attention on the dystonic fingers. Splint-ed fingers should be in positions that are similar to those adopted during normal playing. Eventually, the splints are removed and playing velocity is controlled. Permanent positive goals may be achieved (8). BoNT-A administration may be considered an initial step in a successful rehabilitation programme. This may increase confidence in playing, doctor-patient relations, and articular range of motion, which together may potentiate rehabilitation gains. Administration of BoNT-A may be compared to that of a corticosteroid (CS) injection in an arthritic knee. CS provides knee pain relief, but it will not increase articular stability for long-term pain mitigation.

In conclusion, MFHD is a common, yet often misdiagnosed or late-diagnosed, disease, which it is important that the medical community recognize. Patients often use sensory tricks, which might alert the physician to a possible diagnosis of MFHD. The pathophysiology of musician dystonia remains unclear. Pain might be an important precursor of MFHD; more research is needed on this topic. Although dystonia itself is not inherently painful, it may generate pain through musculoskeletal compensations. BoNT-A is not a definitive treatment for MFHD, but it may enhance other definitive rehabilitation techniques. Further research is needed to determine the long-term effects of BoNT-A on function enhancement in patients with MFHD. Dual-guidance BoNT-A injection may prove useful in detecting specific muscle fibres within the same muscle.

## Supplementary Material












